# Osteonecrosis of the jaws: a review and update in etiology and treatment^☆^

**DOI:** 10.1016/j.bjorl.2017.05.008

**Published:** 2017-06-24

**Authors:** Guilherme H. Ribeiro, Emanuely S. Chrun, Kamile L. Dutra, Filipe I. Daniel, Liliane J. Grando

**Affiliations:** a Universidade Federal de Santa Catarina (UFSC), Programa de Pós-graduação em Odontologia, Florianópolis, SC, Brazil; b Universidade Federal de Santa Catarina (UFSC), Hospital Universitário Polydoro Ernani de São Thiago, Ambulatório de Estomatologia, Florianópolis, SC, Brazil

**Keywords:** Osteoradionecrosis, Osteonecrosis, Therapy, Review, Osteorradionecrose, Osteonecrose, Terapia, Revisão

## Abstract

**Introduction:**

Osteonecrosis of the jaws can result either from radiation, used in radiotherapy for treatment of malignant tumors, or medications used for bone remodeling and anti-angiogenesis such as bisphosphonates. These conditions can be associated with triggering factors such as infection, trauma and decreased vascularity. The management of patients with osteonecrosis of the jaws requires caution since there is no specific treatment that acts isolated and decidedly. However, different treatment modalities can be employed in an associated manner to control and stabilize lesions.

**Objective:**

To review the current knowledge on etiology and management of osteonecrosis of the jaws, both radio-induced and medication-related, aiming to improve knowledge of professionals seeking to improve the quality of life of their patients.

**Methods:**

Literature review in PubMed as well as manual search for relevant publications in reference list of selected articles. Articles in English ranging from 1983 to 2017, which assessed osteonecrosis of the jaws as main objective, were selected and analyzed.

**Results:**

Infections, traumas and decreased vascularity have a triggering role for osteonecrosis of the jaws. Prophylactic and/or stabilizing measures can be employed in association with therapeutic modalities to properly manage osteonecrosis of the jaws patients.

**Conclusion:**

Selecting an appropriate therapy for osteonecrosis of the jaws management based on current literature is a rational decision that can help lead to a proper treatment plan.

## Resumo

**Introdução:**

A osteonecrose da mandíbula pode resultar da radiação utilizada na radioterapia para tratamento de tumores malignos ou medicamentos utilizados para remodelação óssea e antiangiogênese, como os bifosfonatos. Essas condições podem ser associadas a fatores desencadeantes, como infecção, trauma e diminuição da vascularização. O tratamento de pacientes com osteonecrose mandibula requer cautela, pois não existe um tratamento específico que atue de maneira isolada e decisiva. No entanto, diferentes modalidades de tratamento podem ser empregadas de forma associada para controlar e estabilizar lesões.

**Objetivo:**

Revisar os conhecimentos atuais sobre a etiologia e o tratamento da osteonecrose da mandíbula, tanto induzidos por radiação quanto relacionados à medicação, visando melhorar o conhecimento dos profissionais buscando a qualidade de vida de seus pacientes.

**Método:**

Revisão de literatura na base de dados PubMed, bem como pesquisa manual de publicações relevantes na lista de referência de artigos selecionados. Foram selecionados e analisados artigos em inglês publicados de 1983 a 2017, que avaliaram osteonecrose da mandíbula como seu principal objetivo.

**Resultados:**

Infecções, traumas e diminuição da vascularização são fatores desencadeantes da osteonecrose da mandíbula. Medidas profiláticas e/ou estabilizadoras podem ser utilizadas em associação com modalidades terapêuticas para o tratamento adequado de pacientes com osteonecrose mandibular.

**Conclusão:**

Selecionar uma terapia apropriada para o tratamento de osteonecrose da mandíbula com base na literatura atual é uma decisão racional que pode ajudar a estabelecer a um plano de tratamento adequado.

## Introduction

Osteonecrosis was first described as a consequence of ionizing radiation used in the treatment of malignant tumors.^1^ Later, osteonecrosis was discovered as a result of the continued use of some medications from the class of bisphosphonates (BPs)^1^, ^2^ and more recently as a result of the use of drugs that act on bone remodeling and anti-angiogenesis.^3^

Radio-induced osteonecrosis is called osteoradionecrosis (ORN), and is defined as exposure of necrotic bone that persists for over three months in a previously irradiated area receiving ionizing radiation above 50 Gy and is not caused by tumor recurrence.^4^

In turn, medication-related osteonecrosis of the jaws (MRONJ) is also defined clinically by exposure of necrotic bone, but the following characteristics should also be present: (a) patient should be in treatment or have undergone prior treatment with antiresorptive or antiangiogenic agents; (b) presence of exposed bone, or bone that can be probed via intra and extraoral fistula which persists for more than eight weeks; and (c) no history of radiotherapy (RT) or metastatic lesion evident in jaws.^2^ However, clinical manifestations without bone exposure, such as deep periodontal pocket, loose tooth, trismus, hypoesthesia/numbness of lower lip (Vincent's symptom) and non-odontogenic pain could be either classified as non-exposed MRONJ.^3^

Dentists should be able to act in prevention, early diagnosis and rehabilitation of patients with osteonecrosis of the jaw (ONJ). Therefore, the present article aims to present a concise review regarding etiology and treatment of ONJ, both radio-induced as well as medication-related, to improve professionals seeking improved quality of life of their patients on the basis of the current knowledge.

## Objective and methods

A PubMed search using “osteoradionecrosis”, “osteonecrosis”, “therapy”, “MRONJ”, “jaws” as a term was made from May 1983 to April 2017. Additional papers were included based upon the original literature search and references in the selected papers. Papers about laboratory research, case series, as well as reviews of literature were also included.

### Etiopathogenesis

#### Osteoradionecrosis

Delanian and Lefaix (2004) postulated that ionizing radiation possibly leads to tissue injury by creating a local inflammatory process, in addition to causing the death of osteoblasts and preventing the repopulation of cellular components of bone. These events result in a fibrotic bone with a reduced number of vascularized and viable cells.^5^ This weakened tissue has a high potential risk for developing ORN and the occurrence of minimum chemical or physical trauma can trigger a late inflammatory response that leads to tissue necrosis.^4^

However, ORN may occur spontaneously without local trauma or infection. The high radiation rates, which patients with head and neck cancer are submitted to, are sufficient for the occurrence of bone necrosis.^1^ Thorn et al. (2000) reported 23 cases (29%) of spontaneous ORN, mostly asymptomatic, with only a slight dehiscence of the oral mucosa. Thus, the authors emphasized the importance of identifying early-stage ORN and listed other risk factors such as: trauma by prosthesis, surgery and extraction, 3%, 14% and 55% of cases, respectively.^6^ Moreover, dental implants must be considered a potential risk factor for development of ORN, since the irradiated area undergoes serious cellular and tissue damage. The condition of the host for receiving an implant is not only unfavorable but also contraindicates such invasive procedures.^1^, ^7^

#### Medication-related osteonecrosis of the jaws

The known drug participants in etiology of ONJ are antiresorptive and antiangiogenic agents used in antitumor therapy and for treating in various diseases.^8^ These drugs cause a decrease in bone remodeling capability.

Bone remodeling is a physiological process of balance between deposition (osteoblastic activity) and resorption (osteoclastic activity) of this tissue.^9^ A pathological process sets in when the imbalance between these activities occur. Clinical signs and symptoms include bone necrosis, pain, dysgeusia, bucosinusal communication, foul odor, lockjaw, extraoral fistula, and others.^2^

### Antiresorptive medications

#### Bisphosphonates

BPs are synthetic analog drugs of inorganic pyrophosphate, a compound naturally present in organisms, serving as a physiological regulator of calcification and bone resorption inhibitor.^10^

Four generations of BPs are available (Table 1).^11^ From one generation to another the potential of inhibiting bone resorption evidently increases. The amine grouping exponentially increases the potency of the drug,^12^ leading to suppression of bone regeneration with antiangiogenic properties and activator of T-lymphocytes, resulting in a direct tumoricidal effect.^8^

**Table 1:** Available BP's class medicines.

Generation	Composition	Medicines
1st	Non-nitrogenous	Etidronate
		Clodronate
2nd	Nitrogenous	Pamidronate
		Alendronate
3rd		Olpadronate
		Ibandronate
4th		Rizendronate
		Zolendronate

*Source*: Russel (2007).^12^

These drugs accumulate in the bone matrix and are slowly released over prolonged periods of time, with a half-life of approximately 10 years.^13^ Therefore, they pose risks to development of MRONJ, which is dose-dependent. Even after discontinuation of the drug, risk of developing MRONJ remains.^2^

#### Inhibitor RANK-L

RANK-L is one of the osteoclast activating proteins. Inhibitor RANK-L, in turn, is an antibody preventing the binding of RANK-L to its nuclear receptor, thereby not allowing osteoclastic activity. This inhibition of osteoclast hinders bone regeneration, increases bone density and reduces fracture risk. Drugs with this function, like Denosumab, are used in the treatment of bone disorders such as osteoporosis and bone metastasis of malignant tumors. Nonetheless, these medicines also play an important role in the pathogenesis of ONJ.^14^

MRONJ occurs as an adverse effect dependent on the administered dose of Denosumab as well as BP. However, Denosumab action time is shorter than BPs, making it feasible to treat patients in occurrence of side effects such as ONJ.^3^ The mechanisms of action are different between the drugs, but its effects on bone tissue are similar and the specific characteristics of Denosumab on MRONJ are not yet clear.

#### Antiangiogenic agents

The cellular receptor of vascular endothelial growth factor (VEGF) plays an important role in cancer progression, however, it can be controlled by anti-angiogenic drugs.^15^ These medications, such as Bevacisumab, have antiangiogenic properties favorable to tumor restraint, but on the other hand, can compromise the microvessel integrity. This may lead to injury of bone tissue in addition to preventing the action of VEGF, which may have direct deleterious effects on cell differentiation and bone function and thereby cause a failure in the repair of a physiological trauma, inducing MRONJ.^3^

Few cases of ONJ related to Bevacisumab are described in the literature; patients with early diagnosis of ONJ received conservative treatment or surgery and had a relatively quick response to treatment, but there is insufficient information to enable a comparison with ONJ related to BPs.^16^

#### Dental management of patient with developing risk of ONJ

The natural history of ONJ can evolve in different ways. Lesions can develop spontaneously or after a dental procedure, isolated or recurrent episodes may occur, scarring can occur in a few months or may not be evidenced in a period longer than nine months. It is believed that patients who spontaneously develop ONJ are more likely to have recurrences compared to patients who develop ONJ after a dental procedure.^17^

The AAOMS reported rates of 0.5% risk of developing MRONJ after dental extraction procedures in patients who were administered oral BPs, and rates of 1.6–14.8% risk in patients who use intravenous BPs. The risk of developing MRONJ after other dental procedures such as dental implants, endodontic treatment and periodontal procedures is comparable to the risk associated with tooth extraction.^2^ Some authors indicated implant placement in patients who were administered oral or intravenous BPs as not safe, despite the relatively low risk for MRONJ.^18^

Placement of implants in patients undergoing treatment with BPs less than five years may be considered safe for development of MRONJ, however, osseointegration of implants may be affected by therapy with antiresorptive agents.^19^

Regarding risk of developing ORN after implant placement, Tanaka et al. (2013) sought to assess the impact of irradiation of head and neck rehabilitation therapy with dental implants, stressing that risk factors are potential and multidimensional for the failure of implants in these patients. The benefits of using implant-supported prosthesis rather than the use of conventional dentures must outweigh the risks, and yet the planning must be meticulous.^20^

The various possibilities of the etiology of ONJ taper in a main aggravating factor and the placement of these implants fits as an aggravating factor. The best ways to reduce the risk of ONJ are: (1) professional knowledge about overall health of their patients; (2) strict criteria for dental evaluations in patients eligible for head and neck RT, as well as in patients with antiresorptive and antiangiogenic agents treatment; (3) eliminate all dental infections and improve oral health to prevent future invasive therapies. For patients already being treated with these medications or who have already received ionizing radiation in head and neck, it is suggested that bone manipulation be avoided and combined with close clinical monitoring.^21^

The literature cites development of ORN within the first 12 months post-RT,^22^ 6 months^23^ or immediately after first month of RT. However, later occurrence of ORN is also evidenced after 36 months of irradiation.^24^

Risk-patient identification is the first step in preventing this disease. The medical history taken by dentists did not always cover the data on history of cancers and RT, and professionals did not have a specific management protocol for patients with ONJ.^25^ It is a reminder to health professionals that important attitudes can provide better quality of life for patients and even prevent the development of ONJ.

### Treatments

#### Osteoradionecrosis

Treatments include combination therapies, including antibiotics and corticosteroids, Hiperbaric oxigenation (HBO), bone debridement and surgical resection followed by reconstruction.^1^, ^25^, ^26^

Another alternative consists of two related drugs, pentoxifylline and tocopherol (PENTO), but, used separately, they are unable to reverse radio-induced fibrosis. This association becomes more powerful when associated with clodronate (PENTOCLO).^26^ Different managements of ORN are listed in Table 2.

**Table 2:** Treatments approaches of ORN used in the last 10 years.

Authors/date	*n*	Successful treatments	Follow-up (months)
D'Souza et al. (2007)^24^	23	3 HBO	30 (minimum)
		3 HBO, S	
		5 S	
Coletti and Ord (2008)^27^	19	5 S	18 (mean)
Alam et al. (2009)^23^	33	8 ATB, HBO, S	1–61
		22 ATB, S	
Lee et al. (2009)^28^	13	2 S	6–361
		3 HBO, S	
Oh et al. (2009)^29^	114	4 ATB, HBO	12–382
		7 ATB, S	
		18 ATB, NSD	
		25 ATB, HBO, NSD	
		34 ATB, HBO, S	
Delanian et al. (2011)^30^	54	16 ATB, PENTOCLO	2–36
Hampson et al. (2012)^31^	411	243 HBO	96
Mücke et al. (2013)^32^	94	44 ATB, S	12 (minimum)
Niewald et al. (2013)^33^	11	1 HBO	1–147
		2 NSD	
		5 S	
Lyons et al. (2014)^34^	85	3 ATB, PENTO, DNS	3–60
		4 ATB, PENTOCLO	
		14 ATB, PENTO	
		35 ATB, PENTO, S	
Porcaro et al. (2015)^35^	01	1 ATB, NSD	12
Raguse et al. (2016)^36^	149	2 ATB	27–54
		6 S	
		30 NSD	

ATB, antibiotic therapy; S, surgery; NSD, non-surgical debridement; PENTO, pentoxifylline and tocopherol; PENTOCLO, pentoxifylline, tocoferol and clodronate; HBO, hyperbaric oxygenation.

Table 3 shows in detail possible approaches and treatments of ORN described in the literature in last eleven years. In this data comparison, it can be seen the vast majority of studies include antibiotic therapy (ATB) alone or in combination with another therapeutic modality, being more efficient when associated with bone surgery or debridement. HBO, which results in an increase in tissue oxygen tension and improves collagen synthesis, angiogenesis and epithelization, was evaluated in 9 trials and produced contrasting results, with varying success rates between 0% and 100%. One of the latest treatment options, PENTOCLO, is a well-established protocol since 2005 and the results are amazing, as can be seen in the study of Delanian et al. (2011), where all 54 patients evaluated reached total regression of their lesions in up to 36 months after diagnosis of injury.^29^ The latter seems to be a very promising step in the ORN approach, breaking new ground in management of the disease and allowing its remission.

**Table 3:** Drug therapy described by Delanian et al. (2005) associated with conservative treatment of head and neck cancer patients previously irradiated.

Before starting treatment with PENTOCLO – from 2 to 4 weeks			After starting treatment with PENTOCLO – at least 6 months		
Ciprofloxacin	1 g	1×/day	Pentoxifylline	800 mg	1×/day
Amoxicillin + clavulanate	2 g	1×/day	Tocopherol	1.000 IU	1×/day
Fluconazole	50 mg	1×/day	Clodronate	1.600 mg	5 days/week
Metilprednisolone	16 mg	1×/day	Ciprofloxacin	1 g/day	2 days/week
			Metilprednisolone	16 mg/day	2 days/week

*Source*: Delanian et al. (2005).^37^

Oh et al. (2009)^28^ had no success (0%) in the treatment of ORN in patients treated with surgery alone, while Coletti and Ord (2008)^37^ reached 18% success and Lee et al. (2009)^27^ reached 67% success.

Lee et al. (2009)^27^ treated ORN with HBO associated with surgery, resolving 65% of cases while Alam et al. (2009)^22^ resolved 91%. D'Souza et al. (2007)^23^ stated that HBO associated with surgery does not show statistically different results from results achieved by HBO as an isolated therapy.

One of the most modern approaches in management of ORN includes PENTO. Cure was achieved in 73% of patients who use the drug combination for long-term and 69% short-term^26^ and after six years, Delanian et al. (2011) claimed to have achieved 100% success in treatment of ORN with PENTOCLO.^29^

#### Medication-related osteonecrosis of the jaws

Treatment regimens should include education and consent of patient, routine oral hygiene care to reduce the risk of caries and periodontal disease, use of antibiotics and antimicrobials, regular visits to dentist for reevaluation and preservation of clinical picture with elimination of negative habits (smoking and drinking).^36^

There are different approaches dentists can choose, conducting each case with its own peculiarities in order to stabilize the pathological picture of the patient if complete remission is not possible, which are described in detail in Table 4. ATB is consensus in 95% of reviewed studies and is more effective when combined with others measures, especially bone debridement and/or surgery.

**Table 4:** Treatments approaches of MRONJ used in the last 10 years.

Authors/date	*n*	Successful treatments	Follow-up (months)
Thumbigere-Math et al. (2009)^38^	26	3 ATB, HBO, S	6 (minimum)
		9 ATB, S	
Curi et al. (2011)^39^	25	20 ATB, S, PRP	36 (mean)
Ripamonti et al. (2011)^40^	10	10 ATB, NSD, O_3_	8
Agrillo et al. (2012)^41^	94	57 ATB, NSD, O_3_	6.5 (mean)
Freiberger et al. (2012)^42^	25	7 ATB, S	24
		13 ATB, S, HBO	
Martins et al. (2012)^43^	22	1 ATB	6
		3 ATB, S	
		12 ATB, S, PRP, LLLT	
Schubert et al. (2012)^44^	54	48 S	9
Melea et al. (2014)^45^	38	1 ATB, NSD	6 (minimum)
		2 S	
		7 ATB	
		16 NSD	
Vescovi et al. (2014)^46^	192	17 NSD	6–50
		78 S	
Rugani et al. (2015)^47^	38	2 ATB, S	12
		6 ATB	
		17 ATB, NSD	
Klingelhöffer et al. (2016)^48^	76	22 ATB, S	6–24
Minamisako et al. (2016)^49^	01	1 ATB, NSD, LLLT, PDT	12

ATB, antibiotic therapy; S, surgery; NSD, non-surgical debridement; O_3_, ozonated oil; PENTO, pentoxifylline and tocopherol; HBO, hyperbaric oxygenation; LLLT, low-level laser therapy; PDT, photodynamic therapy; PRP, platelet-rich plasma.

Unlike ORN, protocol with PENTO associated with ATB did not show good results (17%) in healing of MRONJ. In contrast, platelet-rich plasma was also a good treatment alternative, succeeding in over 80% of cases. Low-level laser therapy (LLLT), in turn, was presented as a more efficient approach when combined with ATB and bone debridement. HBO had contrasting results with varying success rates between 25% and 90%.

Regarding MRONJ, it is known the better the oral condition of patient to be subjected to treatment with BPs, the more favorable the prognosis. However, often the patient and attending physician were unaware of the possible oral repercussions that this drug class can cause. And once injury is installed, dentist should make use of the measures recommended by AAOMS to try to solve the disease, such as ATB, mouthwash with 0.12% chlorhexidine gluconate, pain management, bone debridement when needed and infection prevention, as well as keeping up to date on the new effective treatment alternatives that are emerging.^2^

Surgery is the treatment option more adopted for MRONJ.^32^, ^43^ Regardless of whether conservative or extended, it is usually associated with ATB.^42^, ^47^, ^50^ With a varying success rate among cases reported in literature, average treatment success with conservative surgery and extensive surgery are 53% and 67%, respectively. Thus, VELscope system is reported as a promising surgical tool which allows identifying the margin between viable and necrotic bone through bone fluorescence.^48^

Thumbigere-Math et al. (2009)^50^ treated MRONJ with HBO associated with ATB and extensive surgery solving 25% of cases, whereas Freiberger et al. (2012)^41^ solved 52% of cases associating HBO exclusively with ATB.

Therapy performed with platelet-rich plasma associated with ATB has shown good results in patients who are undergoing surgical procedures, achieving a cure rate higher than 80%.^38^, ^42^ Unusual but effective, ozone therapy had a 60.6% and 100% success rate in solving 57 and 10 cases, respectively.^39^

Another therapy that has brought good results in combating MRONJ is LLLT. However, their action is most effective when combined with other therapeutic modalities as well as surgery, platelet-rich plasma and ATB^42^ or associated with non-surgical debridement, ATB and PDT.^49^

## Conclusion

The decision of the best approach for management of ONJ patients, in its different modalities, should always be performed by a multidisciplinary team, considering the general state of the patient and the risks/benefits ratio. Infections, trauma and decreased vascularity have a triggering role both for MRONJ and ORN, which are challenging diseases with no specific treatment that acts alone and resolutely.

Different therapeutic modalities can be employed in an associated manner, such as prophylactic and/or stabilizing measures. Furthermore, continuous updated knowledge of the dental professional is essential for the management of these patients.

## Conflicts of interest

The authors declare no conflicts of interest.
